# Inhibition of CRM1 reverses hypoxia-driven chemoresistance in acute myeloid leukemia via overcoming HIF-1α-mediated lysosomal sequestration

**DOI:** 10.3389/fimmu.2025.1710230

**Published:** 2025-11-27

**Authors:** Xiaohui Feng, Yuntian Ding, Yuling Huang, Hancheng Qin, Xiaobo Wang, Yunxin Zeng

**Affiliations:** 1 Department of Hematology, Guangzhou First People’s Hospital, Institute of Blood Transfusion and Hematology, Guangzhou Medical University, Guangzhou, China; 2 Department of Hematology, The Seventh Affiliated Hospital, Sun Yat-sen University, Shenzhen, China

**Keywords:** CRM1, Selinexor, HIF-1α, lysosomal sequestration, chemoresistance, acute myeloid leukemia

## Abstract

**Introduction:**

In relapsed/refractory acute myeloid leukemia (R/R-AML), hypoxia-driven chemoresistance is orchestrated by HIF-1α-induced P-glycoprotein (P-gp) overexpression and subsequent lysosomal sequestration of anthracyclines. Nuclear-cytoplasmic shuttling of the HIF-1α prolyl-hydroxylase PHD2 is controlled by chromosome region maintenance 1 (CRM1), which is frequently up-regulated in AML; however, whether pharmacologic CRM1 inhibition restores PHD2 nuclear availability to accelerate HIF-1α degradation and reverse chemoresistance remains undefined.

**Methods:**

AML cell lines (MV4–11 and MOLM13) were cultured under normoxic or hypoxic conditions. The effects of hypoxia on drug sensitivity, intracellular drug distribution, and protein expression were assessed using CCK-8 assays, immunofluorescence, Western blot, and flow cytometry. Genetic and pharmacological inhibition of P-gp, HIF-1α, and CRM1 was performed to validate their roles in chemoresistance. Hypoxia-adapted zebrafish CHT xenografts were employed for *in vivo* validation.

**Results:**

Hypoxia reduced AML cell sensitivity to DNR, increased HIF-1α and P-gp expression, and promoted lysosomal sequestration of DNR. Inhibition of P-gp or HIF-1α reversed these effects. CRM1 and PHD2 expression increased under hypoxia, but nuclear accumulation of PHD2 decreased. Selinexor restored PHD2 nuclear localization, promoted HIF-1α degradation, reduced P-gp expression, and enhanced DNR nuclear accumulation. Combination treatment with Selinexor and DNR significantly increased apoptosis and DNA damage *in vitro* and reduced leukemia burden in zebrafish xenografts.

**Conclusions:**

CRM1 inhibition by Selinexor re-establishes nuclear PHD2 residency, increases the degradation of HIF-1α in hypoxia, abrogates P-gp-mediated lysosomal anthracycline trapping, and confers potent *in-vitro* and *in-vivo* chemosensitization. These data provide mechanistic rationale for integrating Selinexor into salvage regimens for R/R-AML.

## Graphical Abstract

**Figure:**
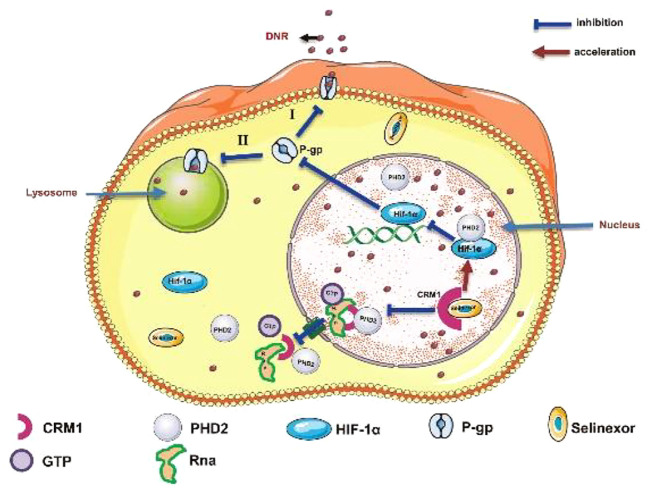
Selinexor overcomes lysosome sequestration-induced drug resistance by inhibiting CRM1, thereby reducing PHD2 nuclear export, enhancing HIF-1α degradation, and ultimately downregulating P-gp expression.

## Background

Despite improvements in the clinical management of acute myeloid leukemia (AML) and the approval of several novel drugs in recent years, the prognosis for relapsed and refractory (R/R) AML patients remains poor, with a 5-year overall survival rate of only 10% (1–3). Chemo resistance as a hallmark of R/R AML mediates the dismal outcomes (4). This highlights the urgent need for new therapeutic strategies to address chemo resistance and improve outcomes for these difficult-to-treat patients.

In hematological malignancies, chemotherapy resistance is closely connected to alterations in the bone marrow (BM) microenvironment, characterized by inflammation, hypoxia, and angiogenesis (4). Regarding hypoxia in the BM microenvironment, the roles of hypoxia-inducible factor-1α (HIF-1α), P-glycoprotein (P-gp), and lysosomal sequestration, which ultimately lead to chemo resistance, are well-documented (5–8). Targeting HIF-1α and P-gp appears promising for anti-leukemia strategies. However, its application to patients with R/R AML has been challenging for several reasons. First, there are no U.S. Food and Drug Administration (FDA)- or European Medicines Agency (EMA)- approved agents for targeting this pathway. Additionally, the potency of anti-leukemia with a single agent may be limited, requiring exploration of combined therapy. Thus, there is a growing demand for new and accessible strategies in clinical practice settings.

The prolyl hydroxylase domain (PHD) family proteins degrade HIF-1α by hydroxylating its proline residues, with PHD2 being the primary enzyme responsible for this process (9, 10). PHD2 is found in both the nucleus and cytoplasm; however, PHD2-mediated hydroxylation of HIF-1α primarily occurs in the nucleus (11, 12). The nucleocytoplasmic shuttling of PHD2 is regulated by chromosome region maintenance 1 (CRM1) (11, 12). Multiple studies have demonstrated that CRM1 is overexpressed in AML. Elevated CRM1 levels in AML cells are closely associated with drug resistance (13–15). Selinexor, a reachable CRM1 inhibitor, has been approved by the FDA for treating relapsed or refractory diffuse large B-cell lymphoma and multiple myeloma (16). The use of Selinexor to reduce chemotherapy resistance in AML has not been fully studied (17).

In the present study, we confirmed that hypoxia-induced upregulation of HIF-1α increases P-gp expression, promotes lysosomal sequestration, and consequently causes drug resistance in AML. We demonstrate that under hypoxia, the expression of both CRM1 and PHD2 increased, while the nuclear accumulation of PHD2 was significantly reduced. Inhibiting CRM1 with Selinexor promoted nuclear accumulation of PHD2, enhanced HIF-1α degradation, subsequently decreased P-glycoprotein expression, and reversed lysosomal sequestration. We provided evidence that the anti-leukemic potency of Selinexor and daunorubicin (DNR) *in vitro* and *in vivo*.

## Results

### Lysosomal sequestration mediated by P-glycoprotein causes chemoresistance in leukemic cells under hypoxia

**Figure 1:**
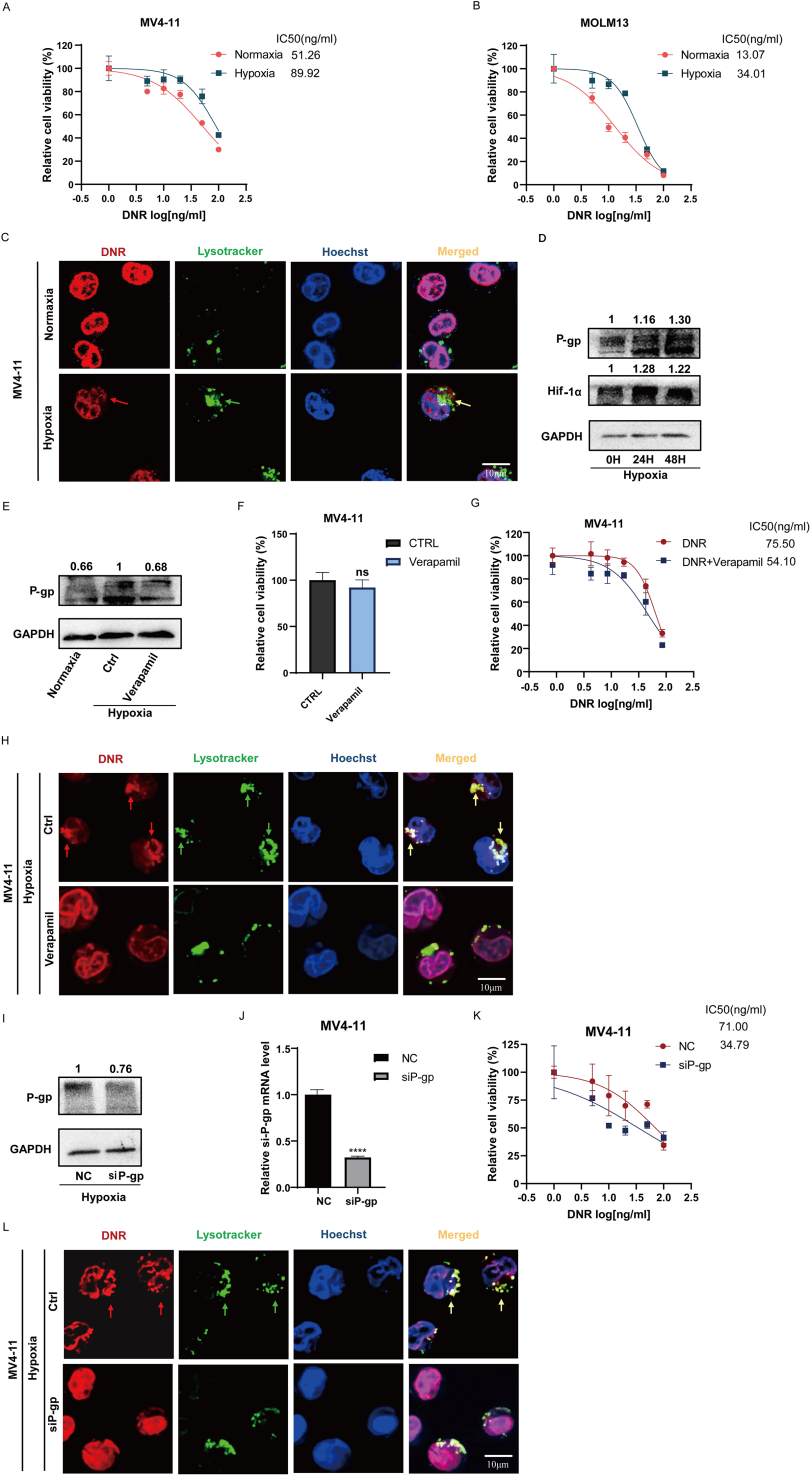
The lysosomal sequestration mediated by P-glycoprotein (P-gp) leads to the chemoresistance of leukemic cells in hypoxia. **(A, B)** Detect the IC50 of DNR in MV4–11 cells in normaxia or hypoxia. **(C)** Observation of DNR intracellular distribution in MV4–11 cells under confocal microscope in normoxic or hypoxic condition. (The “→” indicates lysosome (green fluorescence) sequestrate DNR (red fluorescence), co-localization is visualized as yellow fluorescence.) **(D)** Expression levels of P-gp in MV4–11 cells under hypoxic or normoxic conditions, GAPDH was used as a loading control. **(E, F)** Verapamil (10μM) inhibited P-gp expression in MV4–11 cells without affecting cell viability. **(G)** IC50 of DNR was measured in MV4–11 cells treated with or without verapamil (10μM) in hypoxia. **(H)** Following treatment with verapamil (10μM), lysosomal sequestration of DNR was reduced in MV4–11 cells, resulting in increased nuclear accumulation of DNR. **(I–K)** siRNA was used to knockdown P-gp expression in MV4–11 cells, and DNR IC50 was subsequently determined. **(L)** Confocal microscopy revealed decreased lysosomal sequestration of DNR in siP-gp MV4–11 cells. All experiments were performed at least three independent replicates. * indicates p<0.05, **p<0.01, ***p<0.001, ****p<0.0001.

We first investigated the impact of hypoxia on the chemotherapy sensitivity of leukemia cells. We conducted a comparative analysis to assess the sensitivity of MV4–11 and MOLM13 cells to DNR under both normoxic and hypoxic conditions. The results of relative cell viability experiments showed that the IC50 values of DNR for MV4–11 cells under normoxic and hypoxic conditions were 51.26 ng/mL and 89.92 ng/mL, respectively, while for MOLM13 cells, they were 13.07 ng/mL and 34.01 ng/mL. This indicates that the chemosensitivity of both MV4–11 and MOLM13 cells to DNR decreases in hypoxic conditions (**Figures 1A, B**). An immunofluorescence experiment was conducted to examine the intracellular location of DNR using its inherent red fluorescence. The results showed that both MV4–11 and MOLM13 cells exhibited increased lysosomal biogenesis and enhanced sequestration of DNR, resulting in reduced nuclear import of DNR in hypoxia while more DNR was transported into the nucleus in normaxia (**Figure 1C**, **Supplementary Figure S1A**), aligning with our previous research findings (7). Moreover, Western blot analysis demonstrated an increasing expression of HIF-1α and P-gp in hypoxia-incubated MV4–11 cells (**Figure 1D**).

To further investigate the role of P-gp in lysosomal sequestration, we treated MV4–11 cells with verapamil, a selective inhibitor of P-gp. Experimental concentrations of verapamil (10 μM) reduced the protein expression of P-gp without affecting cell viability (**Figures 1E, F****).** The IC50 value of MV4–11 cells in the DNR combined with verapamil (10 μM) treatment group was lower than that in the DNR-alone treatment group (54.10ng/mL vs 75.5ng/mL) (**Figure 1G****).** We also observed that following treatment with verapamil, DNR was transported into the cell nucleus rather than remaining in the lysosomes (**Figure 1H****).** We subsequently constructed siP-gp MV4–11 for further validation (**Figures 1I, J****).** The IC50 value of DNR for siP-gp MV4–11 cells was 34.79ng/mL, which was lower than that of NC MV4–11 cells (71.00 ng/mL), and the nuclear distribution of DNR in siP-gp MV4–11 cells was observed to increase, whereas lysosomal distribution decreased (**Figures 1K, L**). These findings suggested a reduced capacity of lysosomes in DNR sequestration in siP-gp MV4-11, thereby enhancing the cytotoxic effect of DNR on leukemic cells. The aforementioned results indicate that in hypoxic conditions, the expression of P-gp in leukemic cells is upregulated, thereby promoting the lysosomal sequestration of DNR and subsequently leading to chemoresistance. The inhibition of P-gp has been proven to reverse this phenomenon.

### HIF-1α upregulates P-gp expression and enhances lysosomal sequestration, leading to chemoresistance in MV4–11 cells in hypoxia

**Figure 2:**
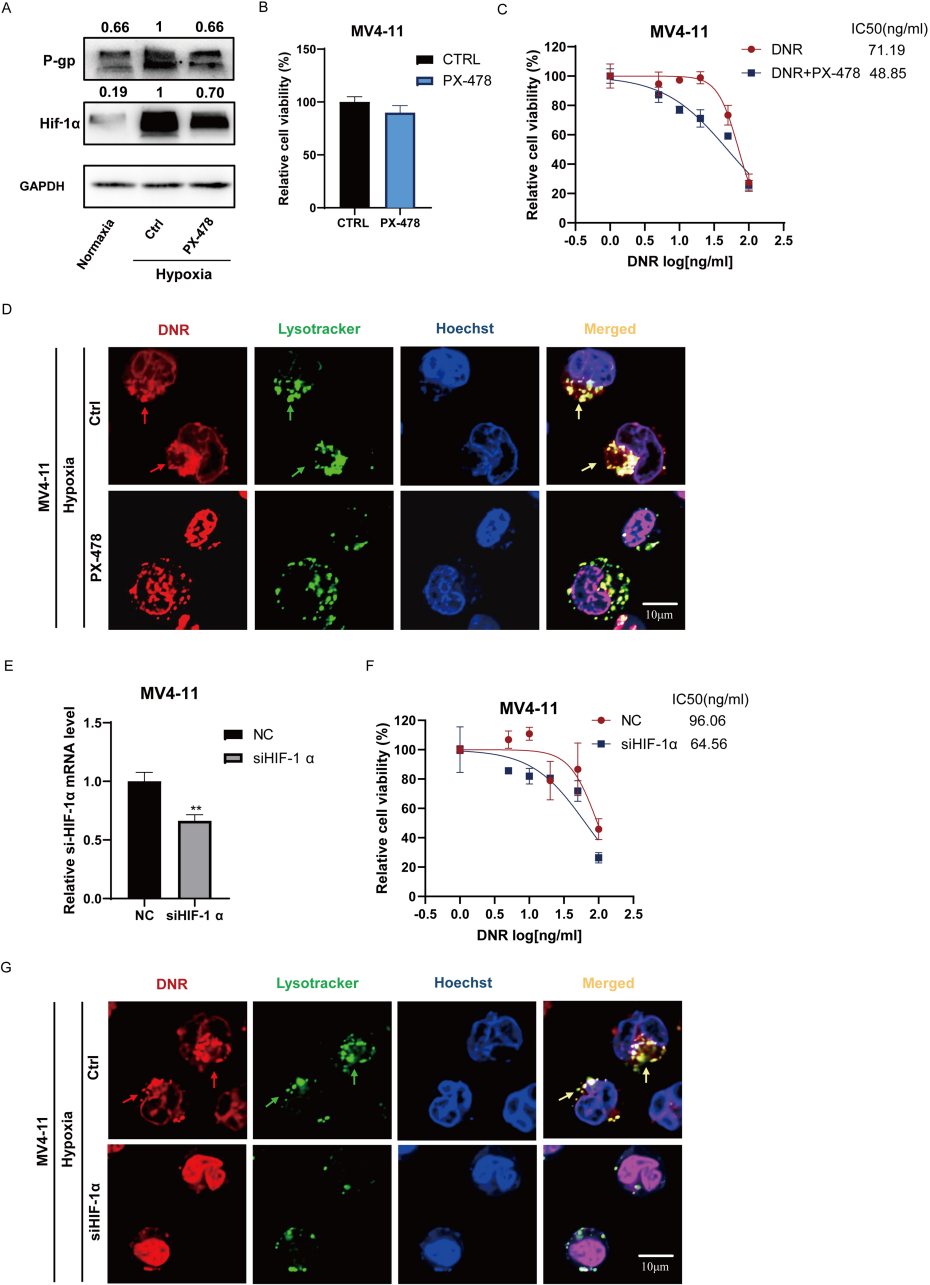
HIF-1α upregulates P-gp expression and enhances lysosomal sequestration, leading to chemoresistance in hypoxia. **(A, B)** PX-478 (10μM) inhibited HIF-1α and P-gp expression in MV4–11 cells without affecting cells viability. **(C)** IC50 of DNR was measured in MV4–11 cells treated with or without PX-478 (10μM) in hypoxia. **(D)** The lysosomal sequestration of DNR in MV4–11 cells with or without PX-478(10μM) pretreatment was observed under confocal microscope. **(E, F)** IC50 of DNR in siHIF-1α MV4–11 and NC MV4-11cells in hypoxia. **(G)** Confocal microscopy revealed lysosomal sequestration of DNR in NC MV4–11 cells and siHIF-1α MV4–11 cells in hypoxia. All experiments were performed at least three independent replicates. * indicates p<0.05, **p<0.01, ***p<0.001, ****p<0.0001.

To investigate the role of HIF-1α in regulating P-gp expression and P-gp-mediated lysosomal sequestration, PX-478, a specific HIF-1α inhibitor, was used both alone and in combination with DNR to treat MV4–11 cells under hypoxic conditions. The changes in cell viability were then observed. PX-478 at a concentration of 10 μM did not affect cells viability while downregulated the expression of both HIF-1α and P-gp proteins in MV4–11 cells in hypoxia (**Figures 2A, B**). This suggests that P-gp is one of the downstream targets of HIF-1α. The IC50 value of DNR for MV4–11 cells in the DNR combined with PX-478 treatment group was lower than that in the DNR-alone treatment group (48.99 ng/mL vs. 71.19 ng/mL), indicating that inhibiting HIF-1α with PX-478 under hypoxic conditions enhanced the chemosensitivity of MV4–11 cells to DNR (**Figure 2C**). Additionally, we found that PX-478 reduced the lysosomal sequestration of DNR in hypoxia (**Figure 2D****).**

We also constructed siHIF-1α MV4-11 (**Figure 2E**). As anticipated, the IC50 values of DNR for siHIF-1α MV4–11 cells were 64.56 ng/mL, which was lower than that of NC MV4–11 cells (96.06 ng/mL) (**Figure 2F**) and alleviated the lysosomal sequestration of DNR in hypoxia (**Figure 2G**). These findings suggested that upregulating HIF-1α in leukemic cells under hypoxic circumstances promoted lysosomal drug sequestration by upregulating the expression of its downstream target P-gp, leading to chemoresistance.

### CRM1 regulates HIF-1α expression by altering the subcellular localization of PHD2 in hypoxia

**Figure 3:**
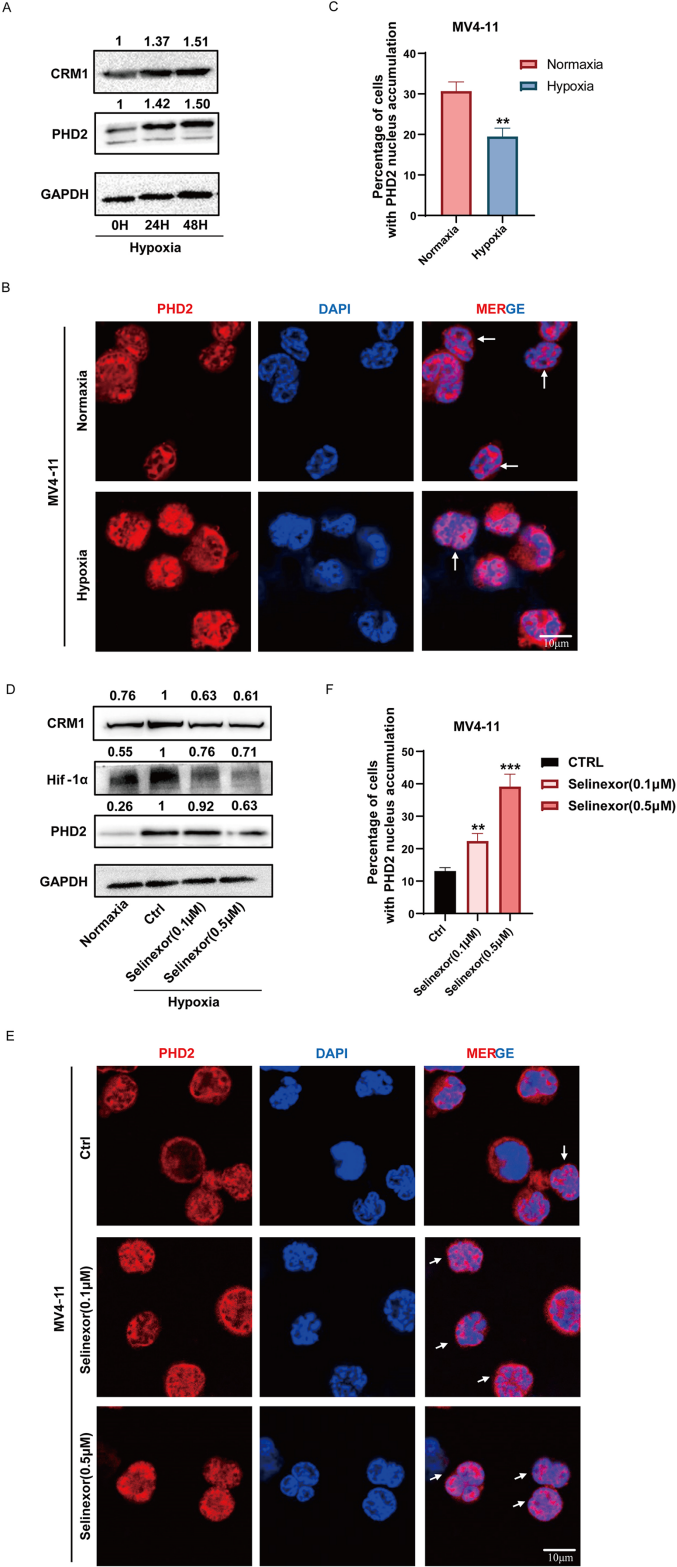
CRM1 regulates HIF-1α by altering the subcellular localization of PHD2 in hypoxia. **(A)** Western blot analysis demonstrated that the expression of PHD2 and CRM1 in MV4–11 cells increased in hypoxia in a time-dependent manner. **(B, C)** Immunofluorescence analysis of PHD2 subcellular localization revealed the proportion of MV4–11 cells exhibiting nuclear accumulation of PHD2 decreased in hypoxia. **(D)** Protein expression levels of PHD2, CRM1, and HIF-1α in MV4–11 cells treated with or without Selinexor(0.1μM, 0.5μM). **(E, F)** Nuclear accumulation of PHD2 in MV4–11 cells treated with or without Selinexor (0.1μM, 0.5μM) in hypoxia. * indicates p<0.05, **p<0.01, ***p<0.001, ****p<0.0001.

Although the inhibitors of HIF-1α and P-gp can overcome lysosomal sequestration-mediated chemoresistance, neither has been used in the clinical treatment of AML. To explore new strategies for overcoming HIF-1α/P-gp-mediated lysosomal sequestration, which leads to chemoresistance, we focused on PHD2, a negative regulator of HIF-1α (9). Nuclear export of PHD2 is mainly regulated by CRM1. Western blot experiments indicated an increased expression of CRM1 and PHD2 in MV4–11 cells under hypoxic conditions, with expression levels gradually accumulating as the duration of hypoxia extends (**Figure 3A**). Immunofluorescence experiments were conducted to investigate differences in subcellular localization of PHD2 in MV4–11 cells incubated in normoxia and hypoxia. We found a reduced proportion of cells with PHD2 nuclear accumulation in hypoxia (**Figures 3B, C**). Subsequently, we treated MV4–11 cells under hypoxic conditions with Selinexor to inhibit the function of CRM1. Western blot analysis demonstrated that the addition of Selinexor reduced the overexpression level of CRM1 in MV4–11 cells under hypoxic conditions, and also decreased the protein levels of PHD2 and HIF-1α, and its inhibitory effect was concentration-dependent (**Figure 3D****).** Immunofluorescence assays demonstrated that the proportion of cells exhibiting nuclear PHD2 accumulation increased when MV4–11 cells were treated with Selinexor, and this increase was concentration-dependent (**Figures 3E, F**). These suggested that the increased expression of CRM1 enhanced the nuclear export of PHD2 in hypoxia. By inhibiting CRM1, Selinexor can promote the nuclear accumulation of PHD2, subsequently enhance the hydroxylation and degradation of HIF-1α, and finally downregulate the expression of Hif-1α.

### Selinexor overcomes lysosomal sequestration and enhances the chemosensitivity of AML cells in hypoxia *in vitro*

**Figure 4:**
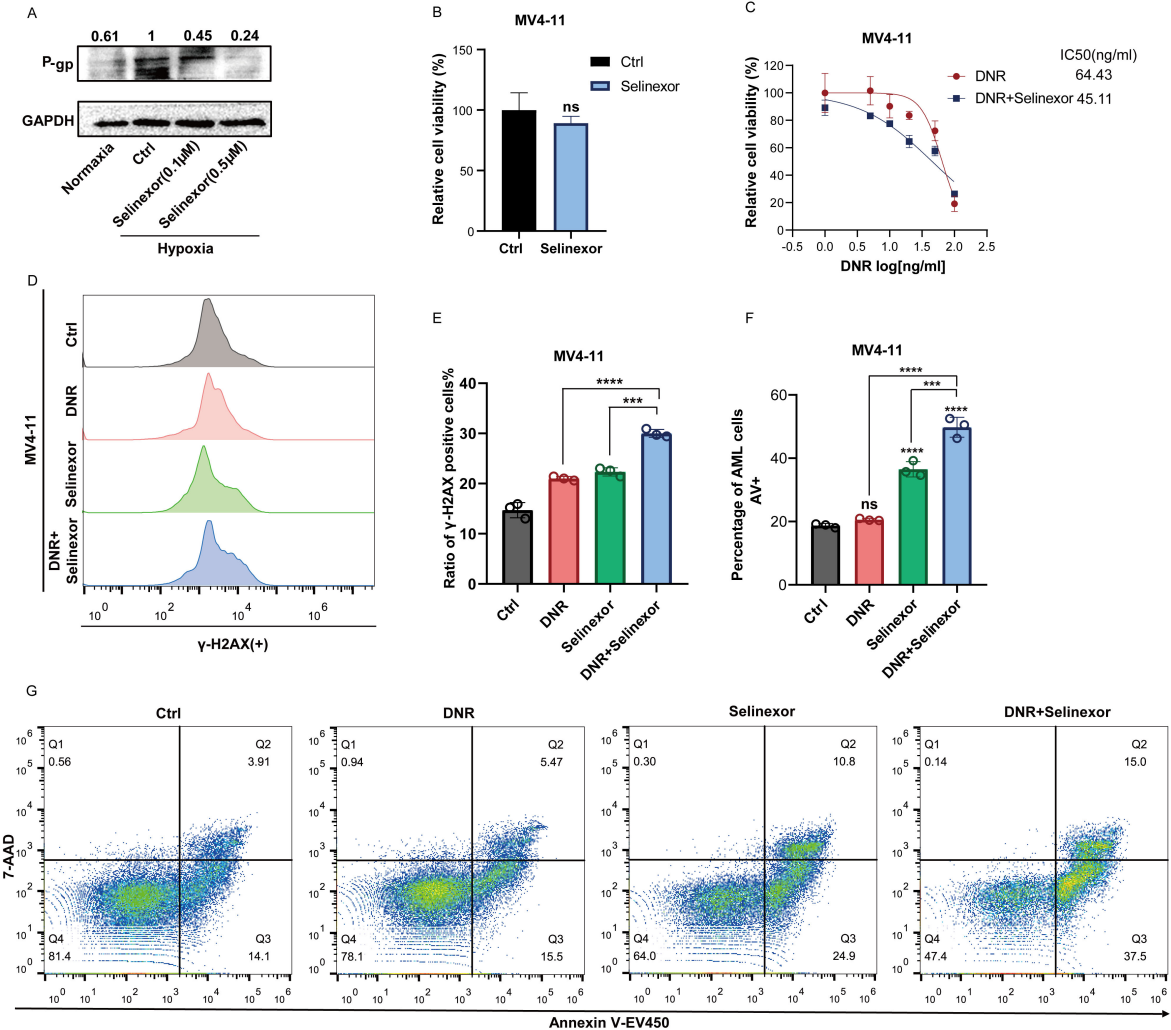
Selinexor overcomed lysosomal sequestration and enhanced the chemosensitivity of AML cells in hypoxia. **(A)** Western blots of p-gp levels in different group cells.MV4–11 in normaxia, MV4–11 in hypoxia and MV4–11 treated with Selinexor at the concentration of 0.1μM or 0.5μM. GAPDH was used as a loading control. **(B, C)** IC50 of DNR was measured in MV4–11 cells treated with or without Selinexor (0.01mM) in hypoxia. **(D, E)** DNA damage graph of Selinexor (0.1μM) and DNR (50ng/ml) in MV4–11 cells. **(F–G)** Apoptosis graph of Selinexor (0.1μM) and DNR (50 ng/ml) in MV4–11 cells. All experiments were performed with at least three independent replicates. * indicates p<0.05, **p<0.01, ***p<0.001, ****p<0.0001.

We investigated the impact of Selinexor on the chemoresistance of leukemic cells in hypoxia. Western blot analysis revealed that Selinexor downregulated the expression of P-gp in MV4–11 cells under hypoxic conditions (**Figure 4A**). When combined with DNR, Selinexor effectively inhibited the expression of P-gp (**Supplementary Figure S1B****).** There was no significant difference in the cytotoxic effect of Selinexor to MV4–11 cells in normoxia or hypoxia (**Supplementary Figure S1C**). Moreover, the viability of MV4–11 cells was not affected after incubation with Selinexor alone at a concentration of 0.05μM. However, when Selinexor (0.05 μM) was given in combination with DNR, the IC50 value for MV4–11 cells was 45.11 ng/mL, which was lower than that in the group treated with DNR alone (64.43 ng/mL)(**Figures 4B, C****).** Flow cytometry was conducted to analyze DNA damage and apoptosis in leukemic cells. When treated with Selinexor in combination with DNR, MV4–11 cells exhibited a higher ratio of γ-H2AX-positive cells than those treated with DNR or Selinexor alone (**Figures 4D, E**). Additionally, apoptosis analysis revealed a higher proportion of apoptotic cells in combination group compared to the control groups treated with either drug individually (**Figures 4F, G**). Immunofluorescence assays showed that DNR was transported into the nucleus when MV4–11 cells were treated by Selinexor (**Supplementary Figure S1D****).** Additionally, we constructed siCRM1 MV4-11, and treated the cells with different concentrations of DNR to assess cell viability. The results indicated that, the IC50 value of DNR in siCRM1 MV4–11 cells was 61.85 ng/mL which was lower than that in NC MV4–11 cells group(IC50 = 72.90 ng/mL) (**Supplementary Figures S1E, F**). These findings suggested that Selinexor can overcome chemoresistance caused by lysosomal sequestration by reducing P-gp expression, exhibiting a synergistic anti-leukemia effect when combined with DNR *in vitro.*

### Selinexor combined with DNR exerts anti-leukemia effects synergistically *in vivo*

**Figure 5:**
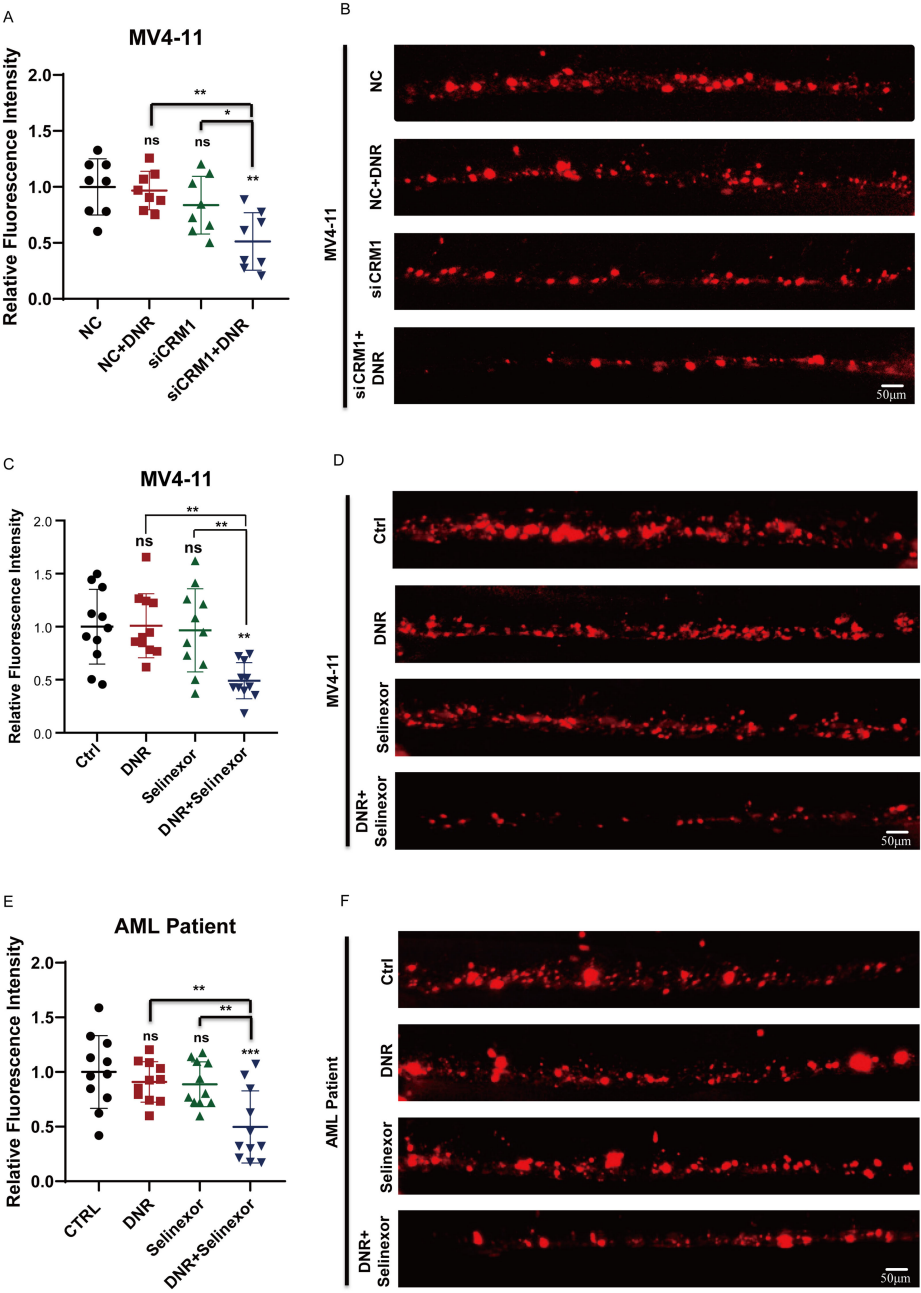
Selinexor combined with DNR exert anti-leukemia effects synergistically *in vivo*. **(A, B)** The zebrafish xenograft models using siCRM1 MV4–11 and NC MV4-11, the xenografted-zebrafish larvae were treated with DNR, and fluorescent intensity of leukemic cells in CHT were counted at two-day post-treatment. **(C, D)** The MV4-11-xenografted-zebrafish larvae were treated with DNR, Selinexor or DNR+Selinexor, and the fluorescent intensity of leukemic cells in CHT were counted at two-day post-treatment. **(E, F)** The zebrafish larvae were xenografted with the leukemic cells from patients and treated with DNR, Selinexor or DNR+Selinexor. The fluorescent intensity of leukemic cells in CHT was counted at two-day post-treatment. * indicates p<0.05, **p<0.01, ***p<0.001, ****p<0.0001.

Our previous study has identified the caudal hematopoietic tissue (CHT), a temporary hematopoietic tissue located in the ventral tail of a zebrafish embryo, which can provide a hypoxic hematopoietic microenvironment and serves as an elegant model for investigating the efficacy of anti-leukemic drugs and the interaction between tumors and the microenvironment *in vivo* (7). We constructed a zebrafish xenograft models using si-CRM1 MV4-11, NC-MV4-11, and leukemia cells extracted from relapsed AML patients, and the fluorescence intensity of leukemia cells in zebrafish CHT was used to evaluate the therapeutic effect *in vivo* (**Supplementary Figure S2****).** We firstly treated the zebrafish embryos with xenografts of NC-MV4–11 and si-CRM1 MV4–11 with DNR to compared the drug sensitivity of leukemia cells at the CHT to DNR. We found that the fluorescence intensity of MV4–11 cells at the CHT site did not reduce significantly upon DNR while that of si-CRM1 MV4–11 decreased significantly (**Figures 5A, B**). Next, zebrafish embryos with xenografts of MV4–11 cells were treated with Selinexor combined with DNR or with either drug individually to observe the therapeutic effect. The results indicated that when Selinexor was used in combination with DNR, leukemia cells at the CHT site of AML zebrafish exhibited a significantly reduced fluorescence intensity compared to the control groups treated with each drug individually. This demonstrates that the combination of these two drugs can effectively eliminate leukemia cells *in vivo* (**Figures 5C, D**). We harvested the same experimental effect in the zebrafish xenograft model constructed by leukemic cells from relapsed AML patients (**Figures 5E, F**). All these results indicate that Selinexor has a synergistic anti-leukemia effect when combined with DNR *in vivo*, potentially offering a treatment strategy for patients with R/R AML.

## Discussion

The outcomes for R/R AML patients remained unfavorable mainly because of chemo resistance. Several studies and our previous research have shown that hypoxia-induced HIF-1α upregulation increases P-gp expression, promotes lysosomal sequestration, and consequently leads to drug resistance in AML, which aligns with our findings in the current study (5–8). However, the unavailability of agents like inhibitors of HIF-1α and P-gp in clinical settings created unmet needs in these patients, highlighting the need for further investigation into the underlying mechanism of the upstream pathway.

This study aims to enhance HIF-1α degradation to reduce drug resistance. The oxygen-sensing HIF-PHDs are key regulators of the degradation of HIF-1α through the hydroxylation of its proline residues, with PHD2 being the primary mediator of this process. Notably, the hydroxylation activity of PHD2 exhibits a localization-dependent manner, and CRM1 controls its nucleocytoplasmic shuttling (9, 11, 12). In this study, we found that CRM1 and PHD2 were upregulated in AML cells, while the concentration of PHD2 in the nucleus is low under hypoxic conditions. Given the phenomena we observed, Selinexor, the inhibitor of CRM1, was administered, and it proved that it could effectively cause the accumulation of PHD2 in the nucleus and decrease the protein levels of PHD2 and HIF-1α in the leukemia cells under hypoxic conditions. Furthermore, we demonstrated that Selinexor decreased P-gp levels and enhanced DNA damage and apoptosis in chemo-resistant leukemic cells under hypoxia. These findings indicate that Selinexor might be a potential way to overcome chemo-resistance in AML.

In clinical practice, increasing the dose of a single agent to achieve better efficacy is not recommended because of the potential for adverse effects. The output of chemo agents might be reduced in conditions with low P-gp expression (18). Therefore, we hypothesized that combination therapy could achieve better efficacy while maintaining a favorable safety profile. We then compared the combination of DNR with Selinexor to DNR and Selinexor alone in terms of killing efficiency at different concentrations. Interestingly, the combination group demonstrates a greater ability to downregulate P-gp and induce apoptosis, with a lower IC50 value. These effects may contribute to DNR by increasing reactive oxygen species (ROS) levels, which could lead to cell death, such as apoptosis, necrosis, and disrupt normal mitochondrial function (18–20). Furthermore, the same results were observed in the zebrafish xenograft model constructed with leukemic cells from relapsed AML patients, which provides compelling evidence for the therapeutic potential of combinatorial therapy with Selinexor and DNR for R/R AML patients.

In conclusion, inhibiting CRM1 with Selinexor promotes the nuclear accumulation of PHD2, which then enhances HIF-1α degradation, decreases P-gp expression, and ultimately overcomes chemoresistance caused by lysosomal drug sequestration. Furthermore, amplification effects were observed when combined with DNR both *in vitro* and *in vivo*, which offer a promising therapy option for R/R AML patients. Of note, the mechanisms demonstrated in established cell lines should be further validated in primary blasts to better assess clinical translatability. Moreover, the potential for serious side effects with this combination necessitates further investigation. Furthermore, using a CRISPR-Cas system to select drug combinations in a well-designed library with AML patient samples would be an effective way to find unbiased, synergistic treatments in the future.

## Materials and methods

### Leukemic cell lines

The leukemic cell lines (MV4–11 and MOLM13) with FLT3-ITD gene mutations were purchased from the Cell Resources Center of Shanghai Institutes for Biological Science, Chinese Academy of Sciences. Cells were cultured in RPMI 1640 (Gibco, USA) containing 10% of FBS (HyClone, USA) and 100 U/ml penicillin and 100 μg/ml streptomycin in an incubator with 5% CO_2_ at 37°C. For hypoxic exposure, leukemia cells were placed in the incubator with 1%O_2_/5%CO_2_/85%N_2_ at 37°C.

### Cell transfection

The small interfering RNAs (siRNAs) for HIF-1α, P-gp, and the corresponding control siRNAs were purchased from IGEbio (China). In brief, MV4–11 cells were seeded in six-well plates (5 × 10^5 cells per well) the night before the transfection procedure. The medium was replaced with Opti‐MEM I Reduced Serum Media (Invitrogen, USA) containing 100 nM siRNA and 10 μL Lipofectamine 2000 (Invitrogen). After 4–6 h, siRNA was removed by replacing the fresh culture medium, and the cells were further incubated for 24 hours. We determined the infection efficiency by analyzing the expression levels of HIF-1α and P-gp using RT-qPCR.

### The cells viability assay

To evaluate the effect of hypoxia on the chemotherapy sensitivity of leukemia cells, MV4–11 cells were incubated in normoxic and hypoxic conditions for 24 hours. Subsequently, 1×10^4 cells were seeded in each well of 96-well plates with medium containing different concentrations of DNR. Finally, cell viability was detected using a CCK-8 kit according to the manufacturer’s instructions. The experiment was performed at least 3 times. The relative cell viability was calculated by dividing the samples by the untreated control.

### Flow cytometry analysis

MV4–11 cells were incubated in an environment with 1% O_2_, 5% CO_2_, and 85% N_2_ at 37°C. After 24 hours, 2×10^5^ MV4–11 cells were seeded into each well of 6-well plates in at least triplicate and supplemented with DNR 50 ng/mL, Selinexor 0.1 μM, or a combination of DNR 50 ng/mL and Selinexor 0.1 μM for an additional 24 hours. The control group consisted of MV4–11 cells treated with dimethyl sulfoxide (DMSO). For cell apoptosis analysis, the cells were incubated for 15 minutes in 500 µL of 1X binding buffer with 5 µL of Annexin V-APC (Elabscience, E-CK-A133) and 5 µL of 7-AAD (Elabscience, E-CK-A133), then analyzed by flow cytometry. For DNA damage analysis, MV4–11 cells were fixed with 70% ethanol overnight at -20 °C and then treated with 50 μl of 1x PBS and 2 μl of antibody (γH2AX-PE) for 30 minutes, followed by analysis using flow cytometry.

### DNR intracellular distribution assay

The MV4–11 cells were seeded in a six-well plate at 1×10^5 cells per well and cultured in a normoxic/hypoxic incubator. The MV4–11 cells in hypoxic incubators were pretreated with DMSO, Verapamil(10μM), PX-478 (10μM), or Selinexor (0.05 μM). After 48 hours, DNR (5μg/ml) was added to the cells. After 4 hours of incubation at 37°C, MV4–11 cells were treated with 5 μg/ml Hoechst 33342 (Solarbio, C0031-1) for 10 minutes, followed by 200 nM LysoTracker Green (Beyotime, C1047S) for an additional 15 minutes. The cells were then rinsed twice with PBS and seeded in confocal dishes for imaging using confocal laser scanning microscopy (Zeiss LSM 800).

### RNA extraction, qPCR

Total RNA was isolated from the leukemia cells using Trizol Reagent according to the manufacturer’s protocol (Invitrogen, America, # 15596026). cDNA was synthesized with First-strand cDNA Synthesis SuperMix (TransGen Biotech, China, # AT341-02), and real-time quantitative PCR (TransStart Green qPCR SuperMix, China, AQ601-02) was performed on a Roche LightCycler 480 II. Primer sequences used were P-gp

F: GGGATGGTCAGTGTTGATGGA

R: GCTATCGTGGTGGCAAACAATA

HIF-1α

F: ATCCATGTGACCATGAGGAAATG

R: TCGGCTAGTTAGGGTACACTTC

house keep genes: β-actin

F: CGAGCAGGAGATGGGAACC

R: CAACGGAAACGCTCATTGC

### Western blot analysis

Pretreated cells were rinsed with ice-cold phosphate-buffered saline. RIPA buffer containing protease inhibitors (1:50, Beyotime, P1008) was used to lyse the cells for 20 minutes at 4°C, followed by centrifuging at 12500g for 20 minutes and collecting the supernatant as the protein extract. After measuring the protein concentration with the BCA kit (CWBio), equal amounts of protein were loaded onto 12% SDS-PAGE gels and transferred to PVDF membranes (Millipore, USA). The membranes were then blocked with 5% bovine serum albumin (BSA) in Tris-buffered saline with Tween (TBST) for 1 hour and incubated with specific primary antibodies: anti-PHD2 antibody (1:1000, NB100-137), anti-HIF-1a antibody (1:1000, Cell Signaling), anti-P-gp antibody (1:1000, Cell Signaling), anti-CRM1 antibody (1:1000, Cell Signaling), and anti-GAPDH (1:1000, Proteintech) at 4°C overnight. Tris buffered saline with Tween (TBST) was used to wash the membranes three times before and after incubating them with the corresponding secondary antibodies for 1 hour at room temperature. Protein bands were detected using the ECL kit (Millipore, WBKLS0500) with an iBrightCL1000 Western Blot Intelligent Imaging System.

### PHD2 subcellular localization assay

MV4–11 cells (1×10^5 cells per well) were seeded in 6-well plates and cultured in normoxic or hypoxic incubators. The MV4–11 cells in hypoxic incubators were treated with DMSO or Selinexor (0.1μM/0.5μM) for 24 hours. Then the cells were collected, dripped onto the slide, and dried in a preheated oven at 90°C to fix the cells on the slide. Then 4% paraformaldehyde (PFA) was used to immobilize cells, followed by permeabilization with 0.5% Triton X-100. The cells were incubated with primary antibody (anti-PHD2 antibody) overnight and with the secondary antibody for 1 hour. Finally, cells were incubated with DAPI for 5min. The cells were sealed for imaging using confocal laser scanning microscopy (Zeiss LSM 800). Calculate the proportion of cells with PHD2 nucleus accumulation in each visual field.

### Patients’ specimens

The specimens from patients with R/R AML analyzed in this study were collected during routine clinical care at the Hematology Department of the 7th Affiliated Hospital of Sun Yat-sen University. The collection and use of these samples were approved by the Institutional Review Board of the 7th Affiliated Hospital of Sun Yat-sen University (approval number KY-2024-059-01), in accordance with international guidelines and the ethical principles outlined in the Declaration of Helsinki. Mononuclear cells were isolated from bone marrow using standard Ficoll-Hypaque gradient density centrifugation and then cryopreserved in aliquots of fetal calf serum with 10% DMSO. Before analysis, 2-3×10^7 cells were thawed and washed twice with RPMI-1640 medium containing 20% FBS, then resuspended in the same medium supplemented with Insulin-Transferrin Selenium (100X, Gibco, 41400045) and GM-CSF (10 ng/ml, PeproTech, 300-03-100ug) at a density of 1×10^6 cells/mL. Both leukemic cell lines and patients’ leukemic cells, before being injected into zebrafish larvae, were labeled with CM-Dil, a lipophilic fluorescent tracking dye (Invitrogen, Catalog# C7000), following the manufacturer’s instructions.

### Leukemia xenografted zebrafish model

Zebrafish (wild-type AB strain) were maintained at 28 °C following the protocol described previously. Embryos were treated with 0.3% Phenylthiourea (PTU, Sigma, P7629-10G) at 10 hours post-fertilization (hpf) to inhibit pigmentation and were anesthetized with 0.090 mg/mL Tricaine (Sigma-Aldrich, A5040-25g) at 48 hpf. Both leukemic cell lines, transfection cells, and patients’ leukemic cells were labeled with CM-Dil, a lipophilic fluorescent tracking dye (Invitrogen, Catalog# C7000), following the manufacturer’s instructions. Approximately 100 to 200 fluorescently labeled cells were injected into the posterior cardinal vein (PVC) of each embryo using a glass capillary needle (Eppendorf, Germany, B100-58-10) controlled by a microinjection system (WPI, USA). The embryos were incubated at 28 °C for recovery before 1 hour post-injection (1hpi), then transferred to 35 °C, where they were kept for the remainder of the experiment. At 1 day post-injection (1 dpi), the injected embryos were selected by counting the number of fluorescent leukemic cells and then treated with Selinexor (0.5 μM), DNR (500 ng/ml), a combination of Selinexor (0.5 μM) + DNR (200 ng/ml), or 0.1% (v/v) DMSO as a negative control. Two days later, the embryos were collected for imaging using a wide-field fluorescence microscope (Olympus IX71). The ImageJ software was used to analyze the fluorescent intensity of leukemic cells. Using a built-in cell counting method with an automated threshold, we identified the leukemic cells to calculate the integrated intensity. By summing all the numbers of the integrated intensity, we get the total fluorescent intensity.

### Statistical analyses

All the experiments were conducted at least in triplicate, and all data are presented as mean ± standard error of the mean (SEM). The statistical significance of differences between groups was assessed using either an unpaired Student’s t-test or ANOVA analysis. P<0.05 was considered statistically significant. * Indicates p<0.05, ** p<0.01, *** p<0.001, **** p<0.0001. Statistical analyses were performed using GraphPad Prism 8.

## Data Availability

The raw data supporting the conclusions of this article will be made available by the authors, without undue reservation.

## Supplementary material

Supplementary Figure 1 **(A)** Observation of DNR intracellular distribution in MOLM13 cells under confocal microscope in normoxic or hypoxic condition. (The “→” indicates lysosome (green fluorescence) sequestrate DNR (red fluorescence), co-localization is visualized as yellow fluorescence). **(B)** Western blots of p-gp levels in different group cells.MV4–11 in normaxia or hypoxia and MV4–11 treated with DNR and/or selinexor. GAPDH was used as a loading control. **(C)** IC50 of Selinexor in MV4–11 cells in normaxia and hypoxia. **(D)** Confocal microscopy revealed lysosomal sequestration of DNR in MV4–11 cells treated with or without Selinexor in hypoxia. **(E, F)** IC50 of DNR in NC MV4–11 cells and siCRM1 MV4-11cells in hypoxia. All experiments were performed at least three independent replicates. * indicates p<0.05, **p<0.01, ***p<0.001, ****p<0.0001.

Supplementary Figure 2 A schematic diagram of the zebrafish xenograft model construction.
