# Supplementary figures and images for: Inhibition of CRM1 reverses hypoxia-driven chemoresistance in acute myeloid leukemia via overcoming HIF-1α-mediated lysosomal sequestration
Source: Front Immunol. 2025 Nov 27;16:1710230. doi: 10.3389/fimmu.2025.1710230 (PMC12696167; doi:10.3389/fimmu.2025.1710230)

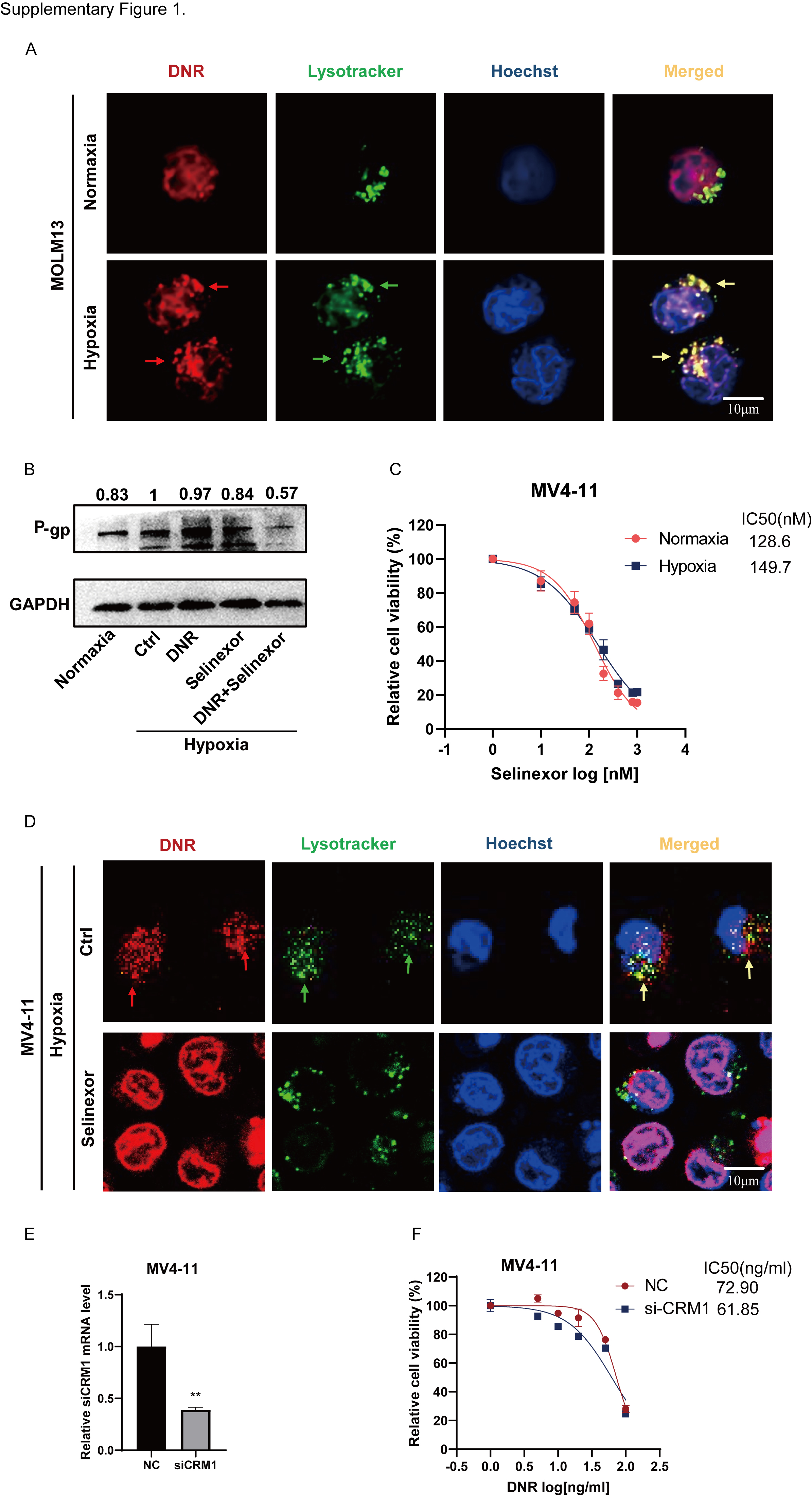

Supplement: Supplementary Figure 1 — (A) Observation of DNR intracellular distribution in MOLM13 cells under confocal microscope in normoxic or hypoxic condition. (The “→” indicates lysosome (green fluorescence) sequestrate DNR (red fluorescence), co-localization is visualized as yellow fluorescence). (B) Western blots of p-gp levels in different group cells.MV4–11 in normaxia or hypoxia and MV4–11 treated with DNR and/or selinexor. GAPDH was used as a loading control. (C) IC50 of Selinexor in MV4–11 cells in normaxia and hypoxia. (D) Confocal microscopy revealed lysosomal sequestration of DNR in MV4–11 cells treated with or without Selinexor in hypoxia. (E, F) IC50 of DNR in NC MV4–11 cells and siCRM1 MV4-11cells in hypoxia. All experiments were performed at least three independent replicates. * indicates p<0.05, **p<0.01, ***p<0.001, ****p<0.0001. [file Image1.jpeg]

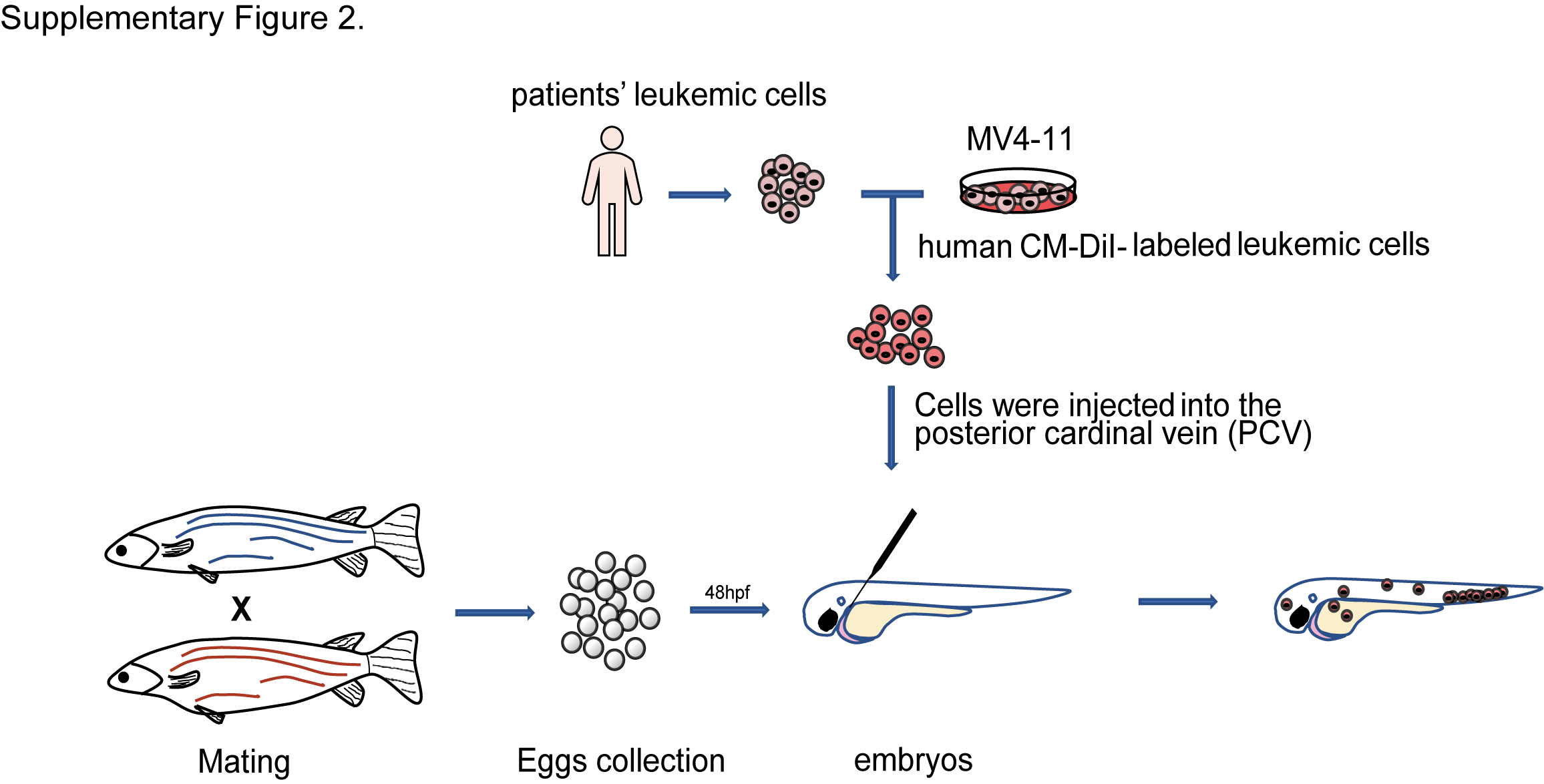

Supplement: Supplementary Figure 2 — A schematic diagram of the zebrafish xenograft model construction. [file Image2.jpeg]
